# Modeling and experimental validation of dynamic defocusing effects in femtosecond laser ablation of complex gear surfaces

**DOI:** 10.1371/journal.pone.0360662

**Published:** 2026-10-13

**Authors:** Wan Li, Hui Xiang, Jiaxia Li, Xingzu Ming, Jun Wang, Rong Wang

**Affiliations:** 1 Hunan Automotive Engineering Vocational University, Zhuzhou, Hunan, China; 2 Hunan University of Technology, Zhuzhou, China; 3 Fuzhou University, Fuzhou, Fujian, China; Northwest Normal University, CHINA

## Abstract

Femtosecond (fs) laser ablation has emerged as a disruptive technology for the ultra-precision finishing of aerospace face gears. However, its application to complex 3D curved topologies is fundamentally bottlenecked by spatially varying nonlinear laser-material interactions. In this study, we propose a comprehensive 3D nonlinear mathematical model for the fs laser ablation of 18Cr2Ni4WA alloy steel to reveal the mechanism of spatial light field distortion. By discretizing the involute face gear topology, the model explicitly couples the localized incidence angle with dynamic defocusing, elliptical beam stretching, and incidence-dependent Fresnel reflection. Theoretical analyses demonstrate that geometrical variations severely distort the energy-coupling efficiency, with the dynamic defocusing distance being directly proportional to the tangent of the incidence angle (tanθ), drastically attenuating the absorbed volumetric energy density in highly inclined regions. Comparison with CNC gear-center measurements yielded descriptive model–measurement differences with a whole-grid MAE of 0.131μm, an RMSE of 0.137μm, and a maximum absolute difference of 0.255μm (6.53% relative difference). These differences were below the expanded measurement uncertainty and therefore are not interpreted as metrological proof of submicrometer resolution. The spatial error pattern is consistent with, but does not directly prove, enhanced plasma-plume shielding within geometrically confined concave valleys. This study provides a model-based foundation for adaptive multi-axis parameter compensation in advanced aerospace manufacturing.

## 1 Introduction

Face gears, characterized by their intersecting or non-intersecting spatial axes, robust load-bearing capacity, and excellent power-split performance, have become pivotal transmission components in advanced aerospace systems, particularly in helicopter main gearboxes [1–3]. The material typically used for these high-performance gears, such as 18Cr2Ni4WA alloy steel, possesses high hardness, exceptional fracture toughness, and fatigue resistance [4]. However, the complex 3D topology of face gear teeth–-featuring varying thicknesses and spatially varying involute profiles–-poses an extreme challenge for precision manufacturing [5]. Traditional mechanical finishing methods, such as CNC grinding and milling, inevitably induce thermal damage, high residual tensile stresses, and severe tool wear, severely limiting the surface integrity and service life of the gears [6,7]. Therefore, there is an urgent need to explore advanced, non-contact finishing technologies capable of achieving micrometer-level precision without compromising the metallurgical integrity of the complex curved surfaces [8].

In recent years, ultrashort pulse lasers, particularly femtosecond (fs) lasers, have emerged as a disruptive technology in the field of ultra-precision manufacturing [9,10]. Characterized by pulse durations significantly shorter than the electron-phonon relaxation time (typically in the order of a few picoseconds), fs lasers achieve a distinct cold ablation regime [11]. According to the Two-Temperature Model (TTM), the extremely high peak intensity of fs lasers leads to nonlinear multiphoton absorption and rapid avalanche ionization, directly vaporizing the target material with minimal heat diffusion into the surrounding bulk [12–14]. This unique physical mechanism effectively minimizes the heat-affected zone (HAZ), prevents micro-cracking, and avoids the formation of severe recast layers, making fs lasers highly attractive for the precision correction of hard-to-machine aerospace alloys [15–17].

Despite the extensive investigation of fs laser ablation on metals, the vast majority of existing theoretical models and experimental studies are confined to flat substrates or simple 2D geometries [18–20]. When transitioning from planar ablation to the precision correction of complex 3D curved surfaces, such as face gears, the interaction between the ultrafast laser beam and the material becomes exceptionally convoluted [21]. The most prominent challenge arises from the spatial variation of the surface normal vector. During the multi-axis scanning of a face gear tooth, the laser incidence angle changes dynamically [22]. According to Fresnel equations, varying incidence angles fundamentally alter the optical reflectivity and energy absorption rates of the material [23–25]. Moreover, an oblique incidence angle distorts the ideal Gaussian circular beam spot into an elliptical profile on the target surface, leading to a highly uneven spatial distribution of laser fluence [26].

More critically, in practical multi-axis laser processing of complex topologies, the ideal kinematic tracking of the focal point onto the curved surface is often physically unattainable due to continuous curvature variations and machine tool trajectory constraints [27]. In this study, *dynamic defocusing* denotes the spatially varying axial separation between the local inclined surface and the Gaussian beam-waist plane within and between illuminated surface patches; it is distinct from a deliberately commanded constant positive or negative focus offset. Defocusing disperses the laser fluence [28]. Furthermore, during fs laser ablation in non-vacuum environments, the generation of high-density plasma plumes may introduce a shielding effect. Under varying defocusing conditions and oblique incidence angles, the expansion dynamics of the plasma plume and its shielding efficiency against the trailing edge of the laser pulse exhibit highly nonlinear behaviors [29,30]. Currently, there is a critical knowledge gap regarding how the dynamic defocusing effect couples with spatial incidence angle variations to govern the final ablation morphology on complex 3D structures. Without a comprehensive mathematical model predicting this coupled nonlinear spatial light field and material removal mechanism, the deterministic precision finishing of face gears using fs lasers remains elusive.

To bridge this gap, this study proposes a novel comprehensive mathematical model for the fs laser ablation of 18Cr2Ni4WA face gears, explicitly incorporating the dynamic defocusing effects and spatially varying incidence angles. The tooth surface is discretized using a spatial meshing method to quantify the localized laser incidence angle at each node. By integrating Fresnel reflection laws, Gaussian beam propagation dynamics, and material absorption coefficients, a 3D nonlinear ablation depth model is established. The theoretical predictions of the ablation morphology are systematically validated against experimental data obtained from a high-precision Klingelnberg gear testing center.

The main contributions of this paper are summarized as follows:

1. A robust 3D nonlinear mathematical model for fs laser ablation is established, coupling the dynamic surface normal vectors with the localized laser fluence distribution.
2. The precise physical relationship governing laser energy absorption of 18Cr2Ni4WA alloy under dynamic defocusing and varying incidence angles is revealed.
3. High-precision spatial mapping and micrometer-level prediction of the face gear topography after fs laser ablation are achieved, providing a theoretical foundation and technological pathway for the ultra-precision correction of complex aerospace transmission components.

## 2 Theoretical modeling of femtosecond laser ablation on complex surfaces

In fs laser processing of complex geometries like face gears, the material removal mechanism cannot be simplified as a 2D planar interaction. The spatial variation of the involute tooth profile induces dynamic changes in both the laser incidence angle and the effective focal position. To accurately predict the ablation morphology, a comprehensive 3D mathematical model coupling the nonlinear spatial light field, dynamic defocusing, and transient material absorption is established in this section.

### 2.1 Ultrafast laser-material interaction and plasma shielding

The ablation of 18Cr2Ni4WA alloy by fs laser pulses fundamentally differs from long-pulse (nanosecond or continuous-wave) laser processing. According to the Two-Temperature Model (TTM), the duration of an fs pulse (~800 fs) is significantly shorter than the electron-phonon thermal relaxation time of the alloy (~1−10 ps). Upon irradiation, the laser energy is primarily absorbed by the free electrons via inverse bremsstrahlung and interband transitions, rapidly elevating the electron temperature to tens of thousands of Kelvin while the lattice remains initially cold. This leads to instantaneous non-equilibrium melting, phase explosion, and direct vaporization of the material with a strictly confined heat-affected zone (HAZ). The conceptual shape of the ablation appearance, characterized by an elliptical spot under non-orthogonal incidence, is illustrated in Fig 1.

**Fig 1:**
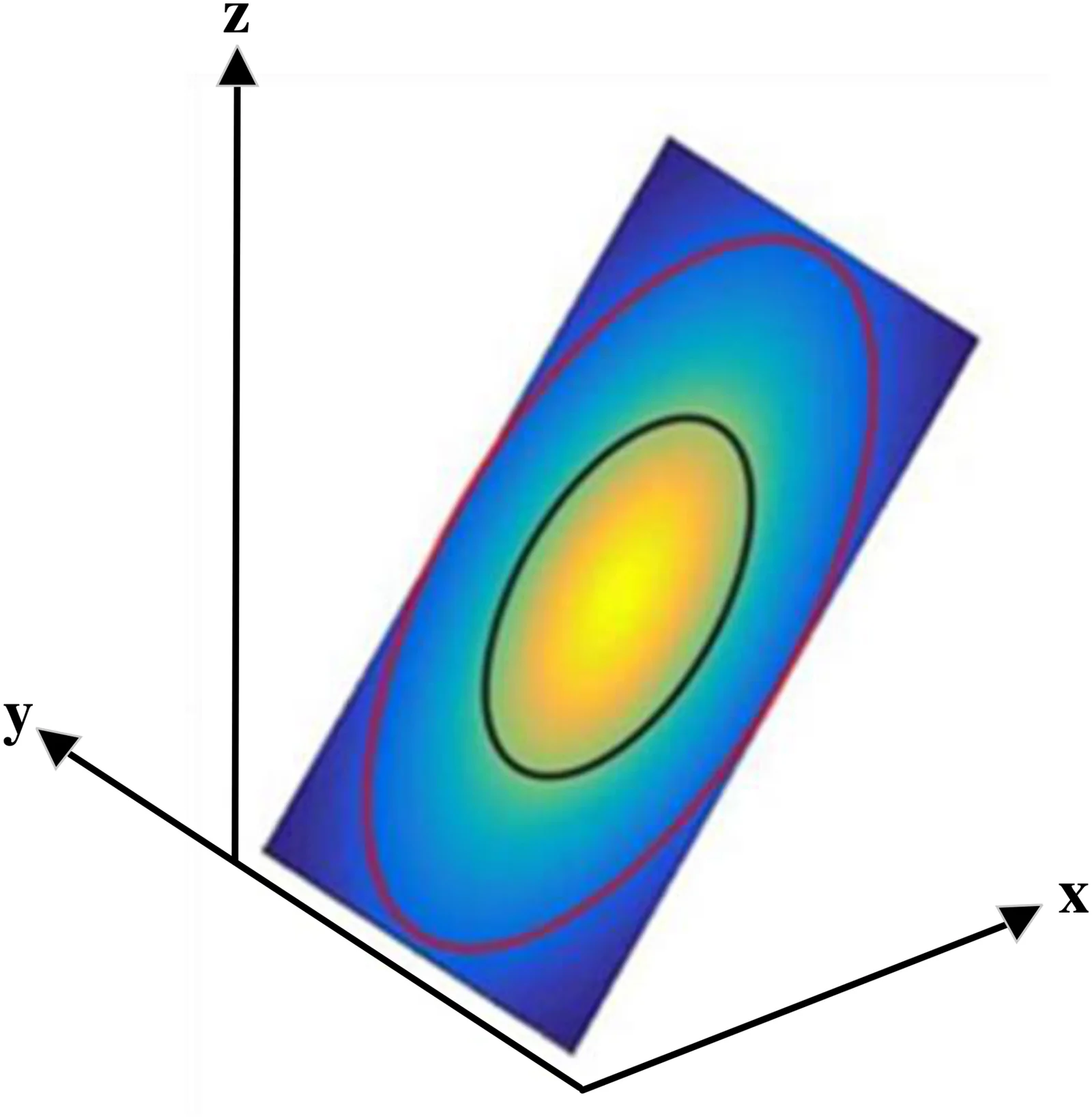
Conceptual diagram of ablation appearance.

Assuming a Gaussian spatial distribution, the incident fluence *F*(*r*) (pulse energy per unit area, J/cm^2^) on a flat plane orthogonal to the beam axis is expressed as:


$$\begin{array}{c} F(r)=F_{0}\exp\left(\frac{-2r^{2}}{\omega_{0}^{2}}\right) \end{array}$$ (1)

where *F*_0_ is the peak fluence at the center of the beam spot, *r* is the radial distance from the beam center, and $\omega_{0}$ is the beam waist radius where the intensity drops to 1/*e*^2^ of the peak value. The propagation behavior of this Gaussian laser energy into the material depth is schematically shown in Fig 2.

**Fig 2:**
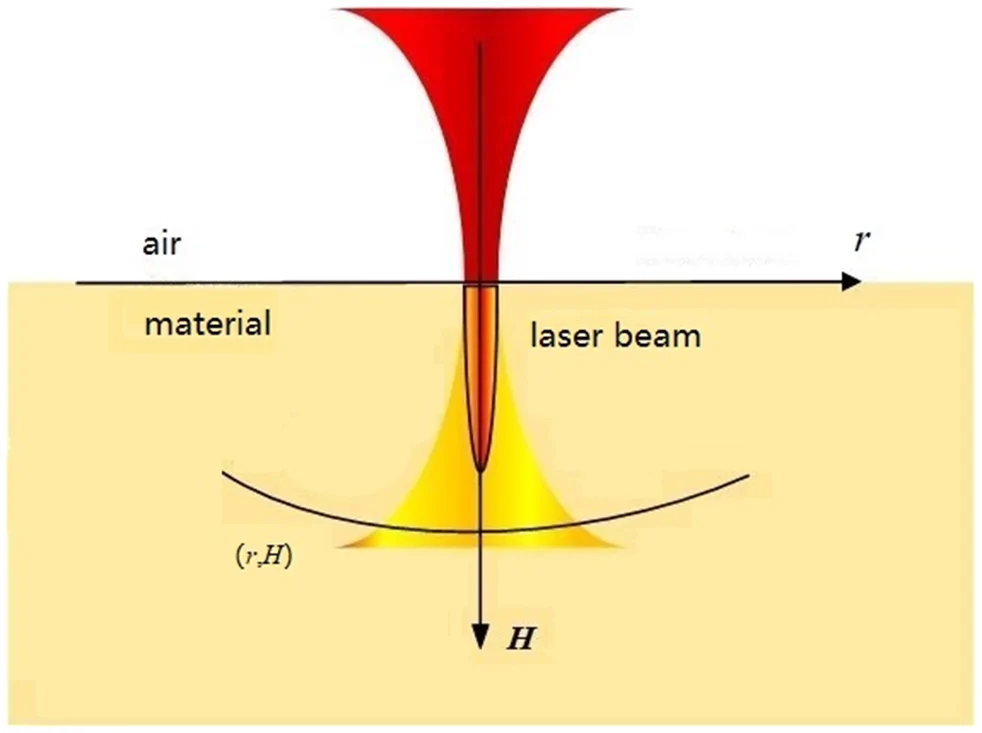
Energy propagation of laser Gaussian beam.

However, in a non-vacuum machining environment, the ejected material and ionized gas form a localized plasma plume over the ablation zone. For complex curved surfaces, geometric confinement may alter plume expansion and attenuation of the trailing portion of the laser pulse. The present model does not solve the plasma dynamics from first principles. Instead, unresolved plasma attenuation and multi-pulse incubation are represented phenomenologically through the process-specific effective threshold $F_{th,eff}$ calibrated under the same scanning conditions as the validation experiment. In the common incubation relation $F_{th}(N)=F_{th}(1)N^{S-1}$, the fitted value corresponds to $F_{th}(N_{eff})$; therefore, *S* and the single-pulse threshold cannot be identified independently from the present fixed-condition dataset.

The model thus contains two distinct levels. Gaussian-beam propagation, local-surface geometry, elliptical footprint formation, Fresnel reflection, and Beer–Lambert attenuation are physics-based components. The calibrated effective threshold is an empirical component that incorporates unresolved cumulative and plasma-related losses. Consequently, residual-error patterns can be interpreted as being consistent with plasma shielding, but cannot establish that mechanism without direct plasma diagnostics.

### 2.2 Spatial energy distribution and dynamic defocusing effect

When the fs laser beam scans across the face gear tooth, the spatially varying surface normal dictates the local incidence angle θ. Unlike orthogonal irradiation where the beam footprint is circular, an oblique incidence angle stretches the beam into an elliptical profile on the tooth surface. Let the ablation zone be defined in a local coordinate system (*x*, *y*, *z*). The elliptical spot extends the energy distribution along the *x*-axis (the direction of inclination), mathematically modifying the local fluence to:


$$\begin{array}{c} F(x,y,z)=\frac{2P}{\pi \omega_{x}\omega_{y}f}\exp\left(-2\left[\frac{(x\cos\theta {)}^{2}}{\omega_{x}^{2}}+\frac{y^{2}}{\omega_{y}^{2}}\right]\right) \end{array}$$ (2)

where *P* is the average laser power, *f* is the repetition rate, and $\omega_{x},\omega_{y}$ are the effective beam radii along the major and minor axes, respectively.

A far more critical factor in multi-axis machining of 3D curved surfaces is the dynamic defocusing effect defined in the Introduction. The distinct impacts of positive defocusing, zero defocusing, and negative defocusing on the ablation crater morphology are presented in Fig 3.

**Fig 3:**
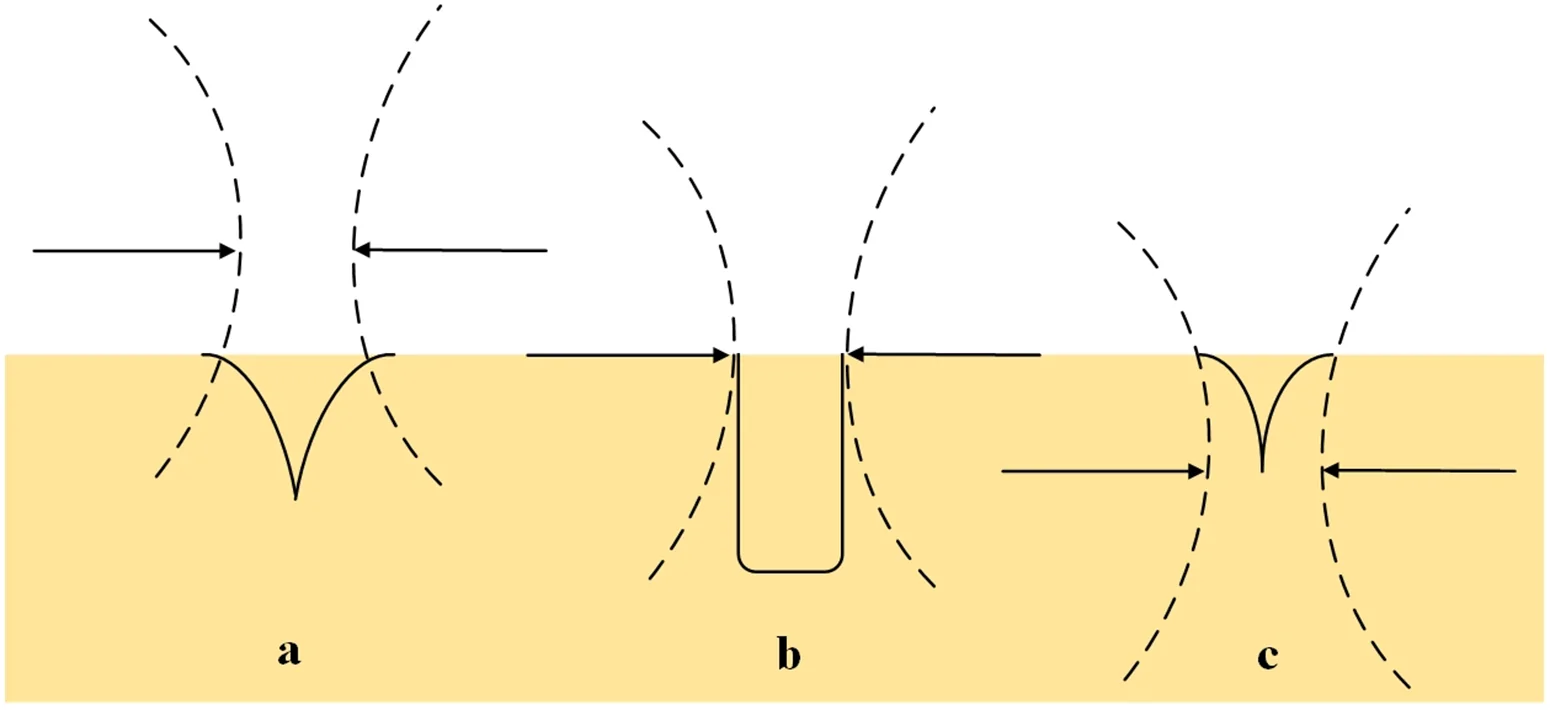
Effect of defocus amount on surface ablation: (a) Positive defocus; (b) No defocus; (c) Negative defocus.

Although the CNC trajectory is programmed to keep the focal point strictly on the surface mesh nodes, the local inclination θ inherently splits the material surface relative to the beam waist. Based on the propagation dynamics of Gaussian beams, the cross-sectional beam radius ω(Δf) at a specific defocusing distance Δf expands hyperbolically, as illustrated in Fig 4.

**Fig 4:**
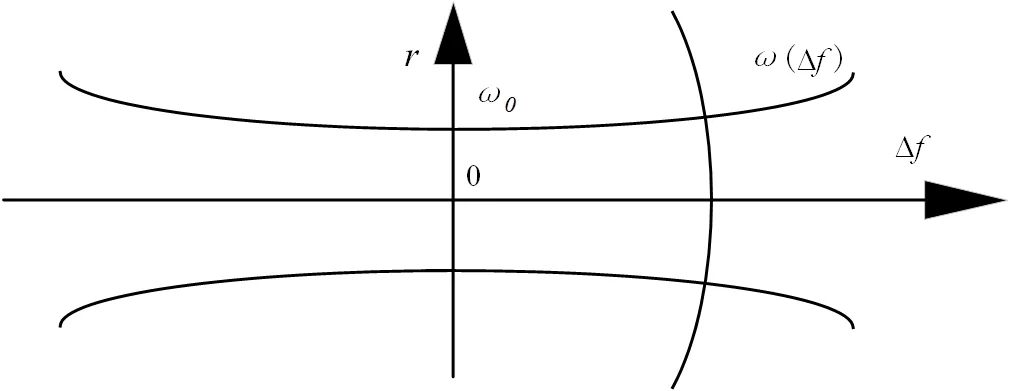
Schematic diagram of changes of beam radius.

The governing equation for this expansion is:


$$\begin{array}{c} \omega (\Delta f)=\omega_{0}\sqrt{1+{\left(\frac{\lambda \Delta f}{\pi \omega_{0}^{2}}\right)}^{2}} \end{array}$$ (3)

where λ is the laser wavelength and $z_{R}=\pi \omega_{0}^{2}/\lambda$ is the Rayleigh range. For a local coordinate system centered at the programmed focal point, let the beam propagate along *z* and let the tangent plane be tilted by θ about *y*. The local plane is z=xtanθ; hence, a point displaced by *x* along the projected inclination direction has the axial offset (Fig 5)


$$\begin{array}{c} \Delta f=x\tan\theta \end{array}$$ (4)

**Fig 5:**
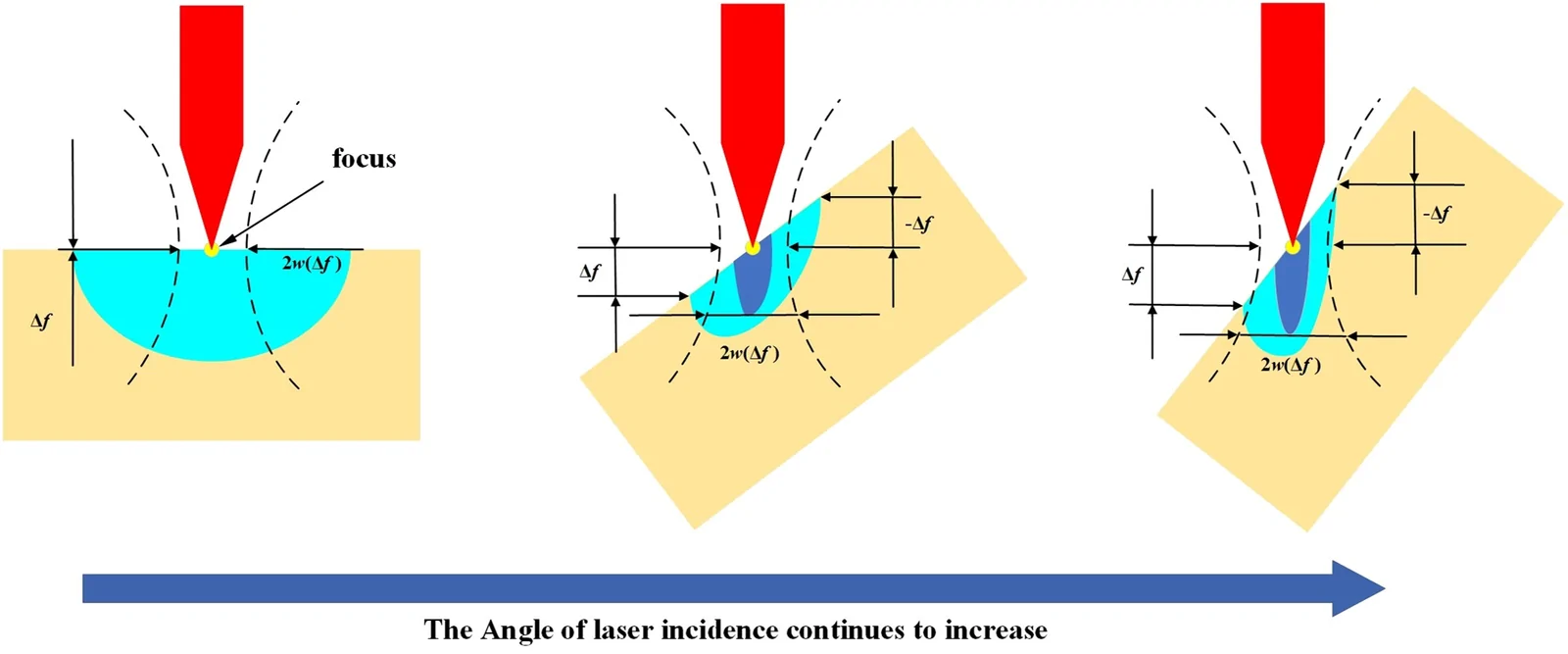
Effect of incident angle on defocus during femtosecond laser precision correction of tooth surface.

Substituting this geometrical relation into the Gaussian beam propagation equation yields the modified spatially variant local fluence Q(x,θ) under dynamic defocusing:


$$\begin{array}{c} Q(x,\theta )=\frac{F_{0}}{1+{\left(\frac{\lambda x\tan\theta }{\pi \omega_{0}^{2}}\right)}^{2}}\exp\left(\frac{-2x^{2}\cos^{2}\theta }{\omega_{0}^{2}\left[1+{\left(\frac{\lambda x\tan\theta }{\pi \omega_{0}^{2}}\right)}^{2}\right]}\right) \end{array}$$ (5)

This equation reveals that an increased incidence angle θ not only geometrically elongates the beam footprint but also exacerbates the fluence dispersion caused by dynamic defocusing, reducing the peak fluence at the extremities of the ablation crater. The relation Δf=xtanθ is applied locally rather than treating the entire curved gear surface as a plane. After discretization, a tangent coordinate system is constructed at the central node of each illuminated patch. In local principal-curvature coordinates, the surface can be expanded to second order as


$$\begin{array}{c} z(x,y)=x\tan\theta +\frac{1}{2}\left(\frac{x^{2}}{R_{1}}+\frac{y^{2}}{R_{2}}\right)+O(\rho^{3}), \end{array}$$

where *R*_1_ and *R*_2_ are the local principal radii of curvature and $\rho =\sqrt{x^{2}+y^{2}}$. The adopted expression retains the first-order tangent-plane term. It is valid when the illuminated-patch dimensions $w_{x}$ and $w_{y}$ are much smaller than the corresponding curvature radii, $\max(w_{x}/R_{1},w_{y}/R_{2})\ll 1$, the curvature-induced sag $w^{2}/(2R_{\min})$ is much smaller than the Rayleigh range $z_{R}$ (or the allowable axial-defocus scale), the paraxial Gaussian-beam approximation holds, and the beam axis is effectively fixed during one local exposure. Higher-order curvature effects become significant near regions with small local curvature radii or when the spot/patch size is increased such that these scale-separation conditions are no longer satisfied; the quadratic curvature terms must then be retained and the fluence evaluated directly on the curved surface.

### 2.3 Incidence-angle-dependent optical reflection and absorption

Before the laser energy can induce ablation, it must couple into the 18Cr2Ni4WA alloy surface. The optical reflectivity *R* of the metallic surface is highly sensitive to the incidence angle θ and polarization state. The initially linearly polarized FemtoYL-100 output was converted to circular polarization by a zero-order quarter-wave plate immediately before the galvanometer scanner. Therefore, the total reflectivity is the average of the s-polarized ($R_{s}$, perpendicular) and p-polarized ($R_{p}$, parallel) reflectance components governed by the Fresnel equations:


$$\begin{array}{c} R_{s}={\left|\frac{\cos\theta -(n+i\kappa )\cos(\arcsin(\frac{\sin\theta }{n+i\kappa }))}{\cos\theta +(n+i\kappa )\cos(\arcsin(\frac{\sin\theta }{n+i\kappa }))}\right|}^{2} \end{array}$$ (6)

$$\begin{array}{c} R_{p}={\left|\frac{\left(n+i\kappa )\cos\theta -\cos(\arcsin(\frac{\sin\theta }{n+i\kappa })\right)}{\left(n+i\kappa )\cos\theta +\cos(\arcsin(\frac{\sin\theta }{n+i\kappa })\right)}\right|}^{2} \end{array}$$ (7)

$$\begin{array}{c} R(\theta )=\frac{1}{2}(R_{s}+R_{p}) \end{array}$$ (8)

where *n* and κ are the refractive index and extinction coefficient of the 18Cr2Ni4WA alloy at the 1030 nm wavelength, respectively. Consequently, the actual energy absorptivity of the material is β(θ)=1−R(θ). As θ increases beyond a critical angle, R(θ) sharply increases towards unity (Fig 6), meaning a significant portion of the incident energy is reflected away and lost.

**Fig 6:**
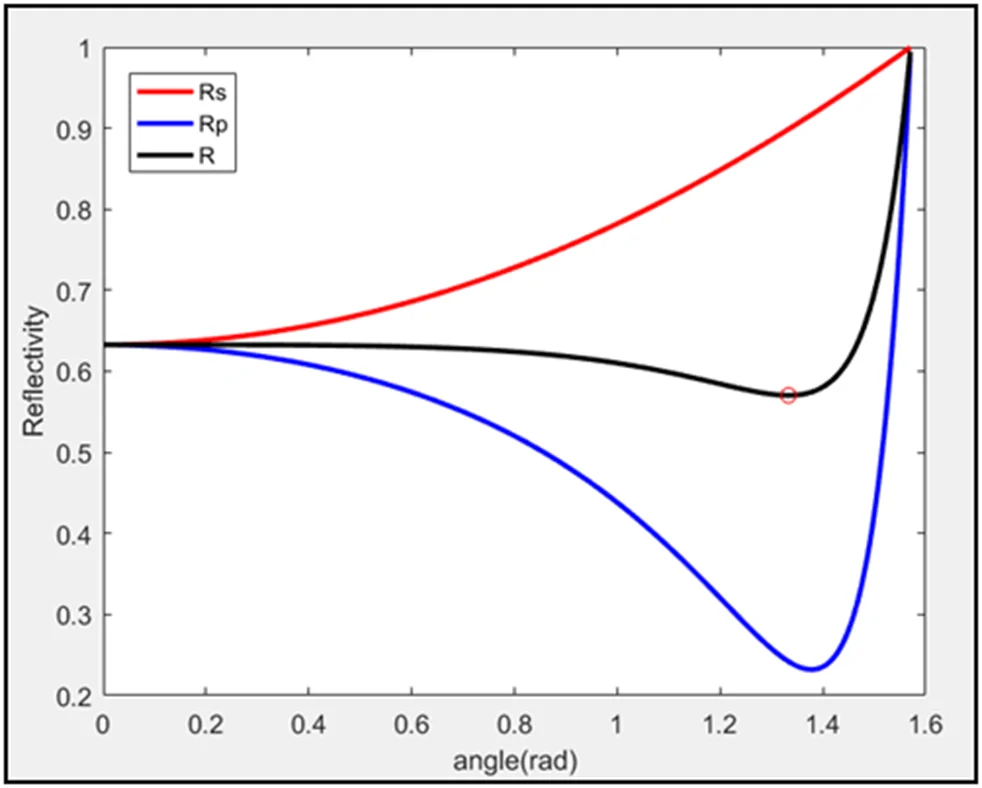
Trend diagram of laser reflectivity with incident angle variation.

Following the initial surface reflection, the absorbed fluence penetrates the material and undergoes exponential attenuation along the depth direction *H*, conforming to the Beer–Lambert law. The absorbed volumetric energy density $q_{v}(H,r)$ is


$$\begin{array}{c} q_{v}(H,r)=\beta (\theta )\alpha F(r)\exp(-\alpha H) \end{array}$$ (9)

where $q_{v}$ has units of J/cm^3^, and the absorption coefficient α is related to the extinction coefficient by α=4πκ/λ.

### 2.4 Comprehensive 3d ablation depth model

Ablation occurs when the effective absorbed fluence exceeds the process-specific effective fluence threshold $F_{th,eff}$ (J/cm^2^). By coupling the dynamic-defocusing fluence distribution Q(x,θ), the angle-dependent absorptivity β(θ), and the Beer–Lambert attenuation law, the theoretical ablation depth $Z_{ab}$ along the surface normal at the focal center, under an oblique incidence angle θ, is derived as:


$$\begin{array}{c} Z_{ab}=\frac{1}{\alpha \cos\theta }\ln\left[\frac{(1-R(\theta ))F_{0}\cos\theta }{F_{th,eff}\left[1+{\left(\frac{\lambda \Delta f}{\pi \omega_{0}^{2}}\right)}^{2}\right]}\right] \end{array}$$ (10)

This comprehensive model indicates that the morphology of the fs laser machined crater is the result of a highly nonlinear competition between (1) the geometrical beam stretching, (2) the defocusing-induced intensity decay, and (3) the Fresnel reflection losses at high incidence angles.

## 3 Experimental setup and simulation of morphological evolution

### 3.1 Material preparation and femtosecond laser machining system

The material selected for this study is 18Cr2Ni4WA alloy steel. Prior to laser finishing, the face gear specimens underwent standard CNC high-speed milling, leaving a consistent machining allowance of approximately 4 μm. The initial milled-surface roughness was $R_{a}=0.65\pm 0.08\;\mu$m and $R_{z}=3.2\pm 0.4\;\mu$m. Measurements were obtained on three distinct tooth flanks along the profile direction using a Mahr MarSurf LD 130 profilometer with a cutoff length of 0.8 mm and an evaluation length of 4.0 mm.

The experimental setup employs a customized multi-axis fs laser precision machining center, as schematically depicted in Fig 7. The core light source is an industrial-grade all-fiber femtosecond laser (FemtoYL-100, YSL Photonics), delivering ultrashort pulses with a central wavelength of λ=1030 nm and a pulse duration of τ≈800 fs. A zero-order quarter-wave plate positioned immediately before the galvanometer scanner converted the initially linear output to circular polarization.

**Fig 7:**
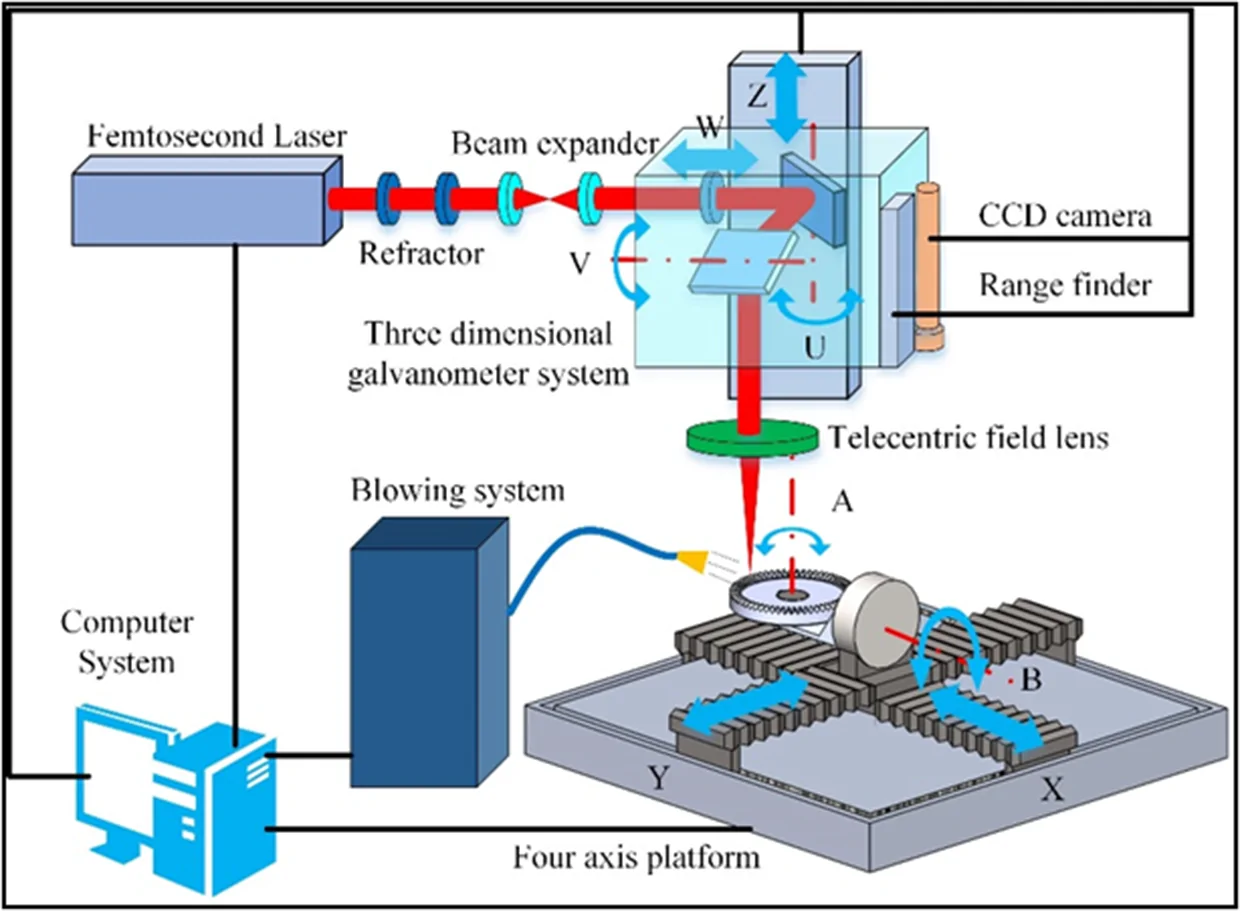
Precision correction system of femtosecond laser for face gear.

Simultaneously, the face gear workpiece was mounted on a high-precision four-axis CNC rotary platform (*X*, *Y*, *A*, *C* axes), allowing the laser focal point to track the spatial involute trajectory. Machining used a 5μm hatch spacing and a bidirectional crossed-raster strategy. The scan direction was rotated by $90^{\circ }$ between successive layers, and 20 passes were applied. The focal position dynamically tracked the mathematical involute-surface nodes through simultaneous four-axis interpolation. The approximately 4μm removal depth reported below is the cumulative depth after this complete multi-pass process.

### 3.2 Topological discretization and optical parameters

Because the normal vector of a face gear tooth changes continuously, a topological spatial discretization strategy was adopted. Utilizing a 3D CAD model, a representative single tooth surface was mathematically meshed into a 5×9 spatial grid (Fig 8). This grid matched the experimentally accessible measurement nodes while sampling the profile and longitudinal directions, including the top, central concave, root, small-end, and big-end regions. A grid-resolution study using 9×17 and 17×33 meshes showed that, relative to the finest mesh, the 5×9 mesh changed the predicted mean depth by 0.5% and the maximum local depth by 1.2%. Thus, the selected grid adequately resolves the macroscopic morphology.

**Fig 8:**
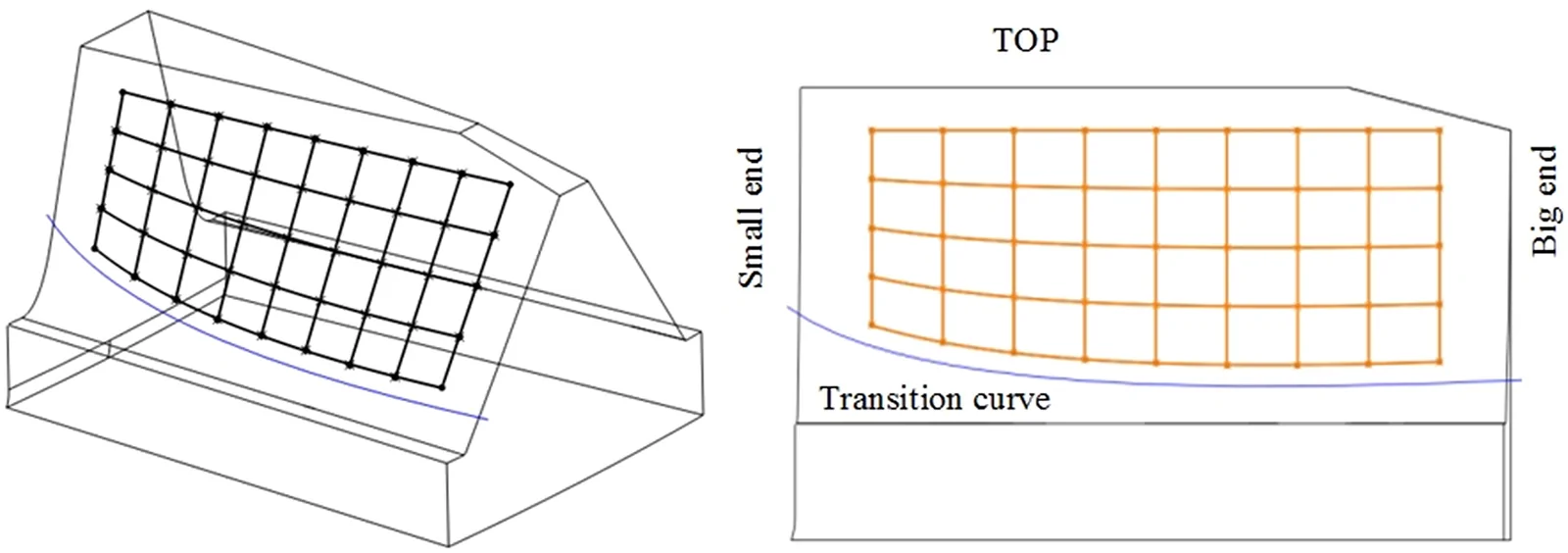
Schematic diagram of meshing and mesh projection of tooth surface.

At each node, the localized spatial normal vector was extracted, and the corresponding laser incidence angle θ was calculated based on the fixed optical axis of the delivery system (Fig 9). The extracted incidence angles exhibited significant spatial heterogeneity, ranging from $37^{\circ }$ near the big end/root region to $73^{\circ }$ near the small end/top region, as detailed in Table 1.

**Fig 9:**
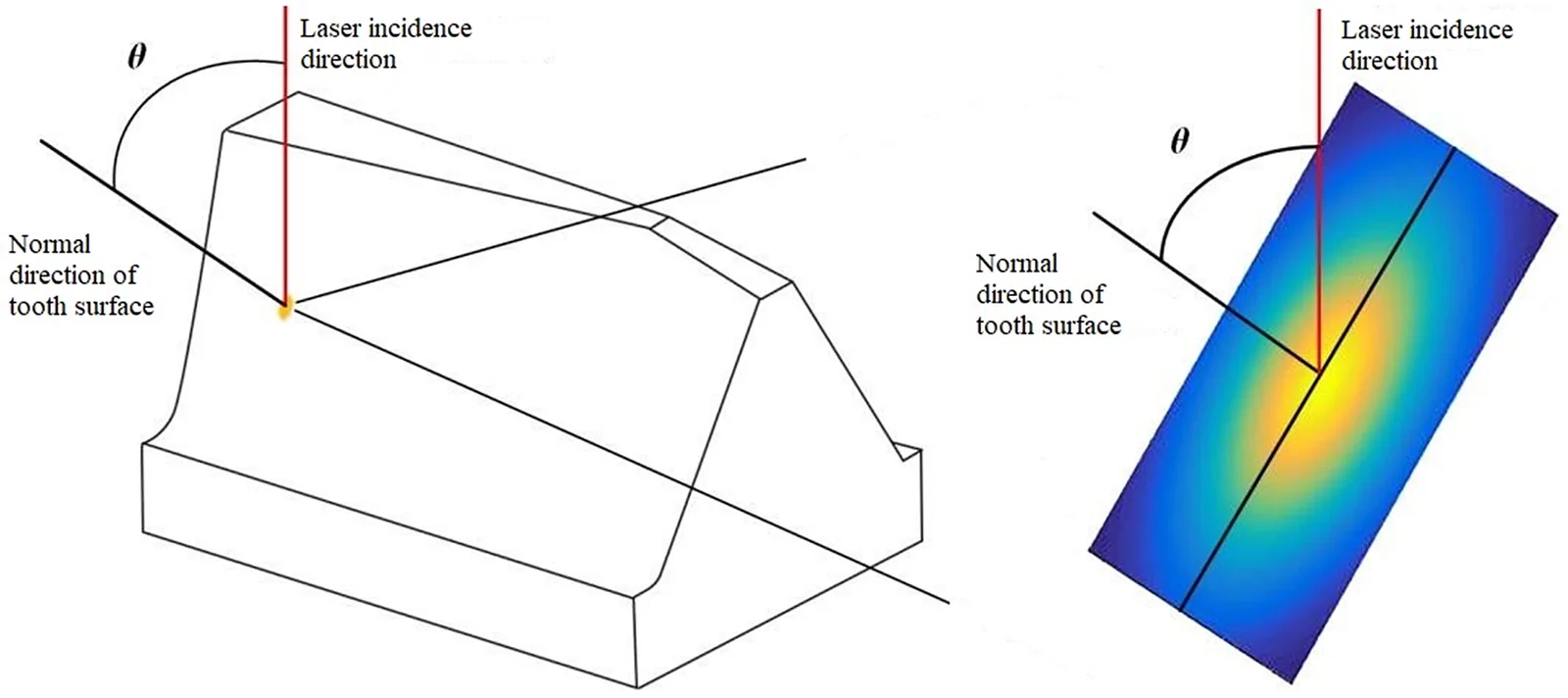
Schematic diagram of measurement method of laser incidence angle.

**Table 1:** Measured laser incidence angles (θ) at the 5×9 spatial grid nodes across the face gear tooth surface (Unit: Degree).

Profile Direction \ Longitudinal	Node 1	Node 2	Node 3	Node 4	Node 5	Node 6	Node 7	Node 8	Node 9 (Big end)
**Row 1 (Top)**	73	71	68	67	66	65	63	62	60
**Row 2**	68	67	65	64	62	63	61	58	57
**Row 3**	64	62	61	62	63	61	60	58	59
**Row 4**	54	54	53	51	52	53	54	52	50
**Row 5 (Root)**	41	39	38	38	39	41	39	39	37

The optical constants were obtained by spectroscopic ellipsometry on a polished 18Cr2Ni4WA reference flat prepared from the same material batch as the gear specimens. Ellipsometric spectra were acquired over 800–2000 nm, and the fitted complex optical constants were evaluated at the 1030 nm processing wavelength, giving a refractive index *n* = 3.192 and an extinction coefficient κ=4.416. These values were used in the s- and p-polarized Fresnel equations, whose average represents the circularly polarized processing beam. The reported *n* and κ should be interpreted as effective optical constants of the polished reference surface. Their uncertainty is influenced by ellipsometer repeatability, optical-model regression and the correlation between *n* and κ, surface preparation and roughness, and possible native-oxide or compositional differences between the reference flat and the processed tooth surface. Because an independent traceable numerical uncertainty budget for *n* and κ was not established, unsupported numerical confidence bounds are not assigned, and the calculated reflectivity is interpreted within this limitation. The effective threshold was calibrated using 5×5 mm flat specimens processed at 65 mm/s and 300 kHz. Depths from 10 repeated measurements were fitted by least squares. The core optical and process parameters and their provenance are listed in Table 2.

**Table 2:** Core parameters for the fs laser simulation and machining.

Parameter	Symbol/Unit	Value	Determination
Central wavelength	λ (nm)	1030	Calibrated laser setting
Pulse duration	τ (fs)	800	Laser setting
Beam waist radius	$\omega_{0}$ (μm)	20	Beam measurement
Average power	*P* (W)	3	Calibrated power setting
Repetition rate	*f* (kHz)	300	Laser setting
Scanning speed	*V* (mm/s)	65	CNC setting
Effective threshold	$F_{th,eff}$ (J/cm^2^)	0.1383	Least-squares calibration, 10 repeats
Optical absorptivity	β	Angle-dependent	Spectroscopic ellipsometry (same-batch polished flat) and Fresnel model
Hatch spacing	— (μm)	5	CNC setting
Scan strategy	—	Crossed raster	Bidirectional; $90^{\circ }$ layer rotation
Number of passes	—	20	CNC setting
Polarization	—	Circular	Zero-order quarter-wave plate

### 3.3 Numerical simulation of dynamic defocusing effects

Based on the comprehensive 3D ablation depth model established in Section 2.4, a numerical simulation was executed using MATLAB to predict the morphological evolution of the ablation craters.

#### 3.3.1 Single-Pulse Morphological Distortion.

The simulation initially focused on the isolated ablation crater morphology. At an ideal orthogonal incidence ($\theta =0^{\circ }$), the simulated crater exhibits an axisymmetric shape (Fig 10). However, as the incidence angle increases, the 3D model vividly demonstrates a dual-distortion effect where the crater opening becomes distinctly elliptical (Fig 11).

**Fig 10:**
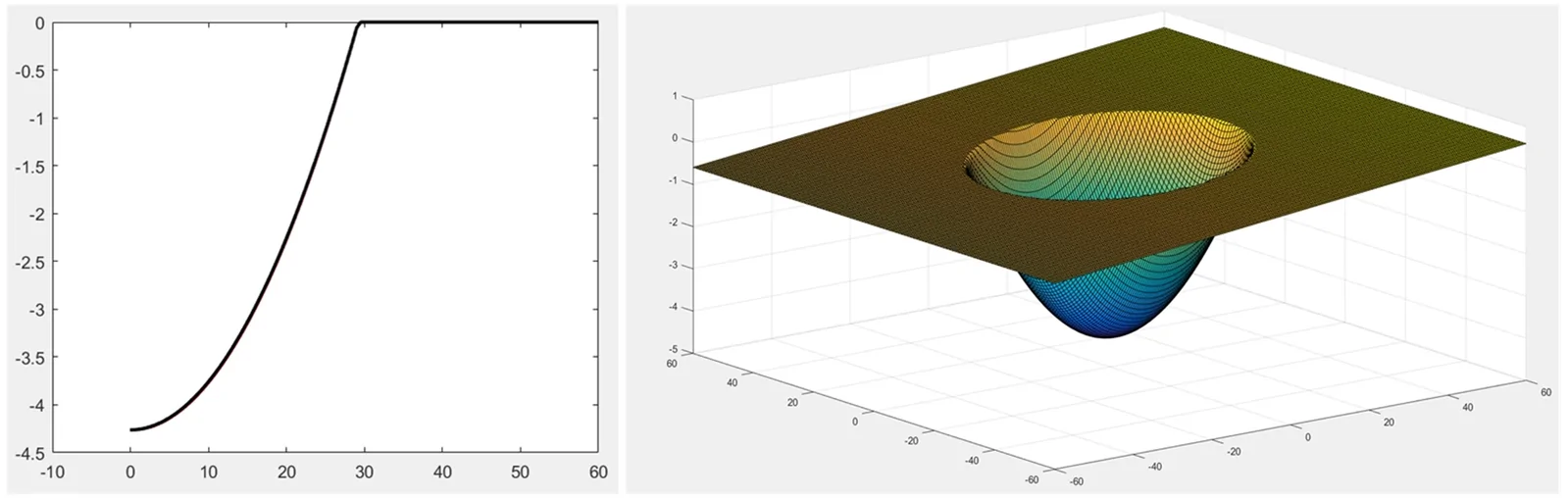
Depth values of single node ablation pits and 3D model under orthogonal incidence.

**Fig 11:**
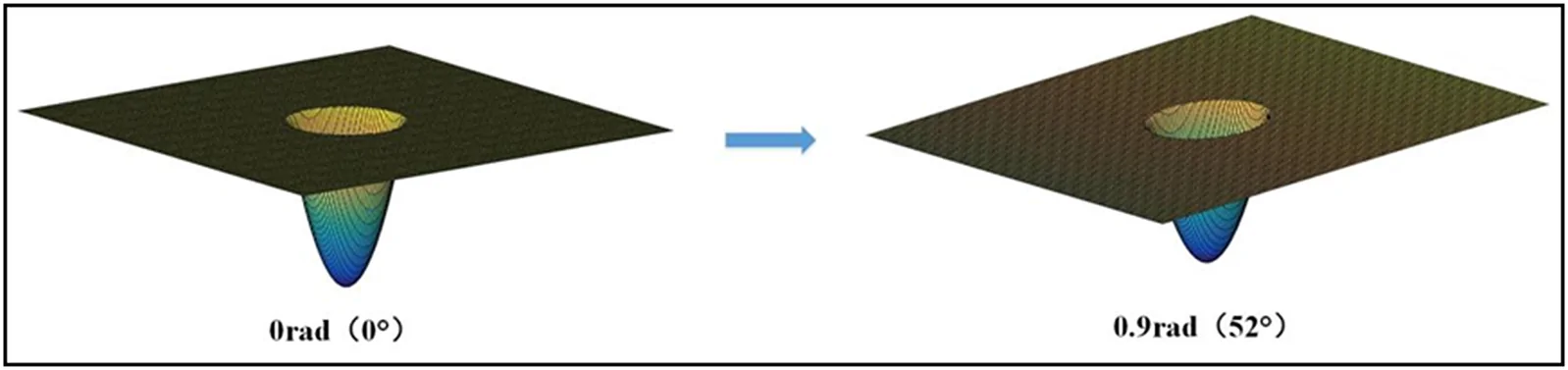
3D model of ablation pits on tooth surface varying with laser incidence angle.

#### 3.3.2 Ablation depth vs. incidence angle.

The simulated trend and experimental node values both show a pronounced negative association between cumulative ablation depth and incidence angle (Fig 12). Over the investigated range of $37^{\circ }$–$73^{\circ }$, least-squares fitting of all 45 experimental nodes gave the negative-slope relation $d\;(\mu m)=(-0.0200)\theta \;({\;}^{\circ })+5.178$, with *R*^2^ = 0.738. Thus, the fitted slope coefficient is explicitly −0.0200. The 95% confidence interval for the slope was [−0.0237,−0.0163] and *p* < 0.001. This linear fit is descriptive only over the investigated angular interval; the underlying physical model is nonlinear and should not be extrapolated beyond this range.

**Fig 12:**
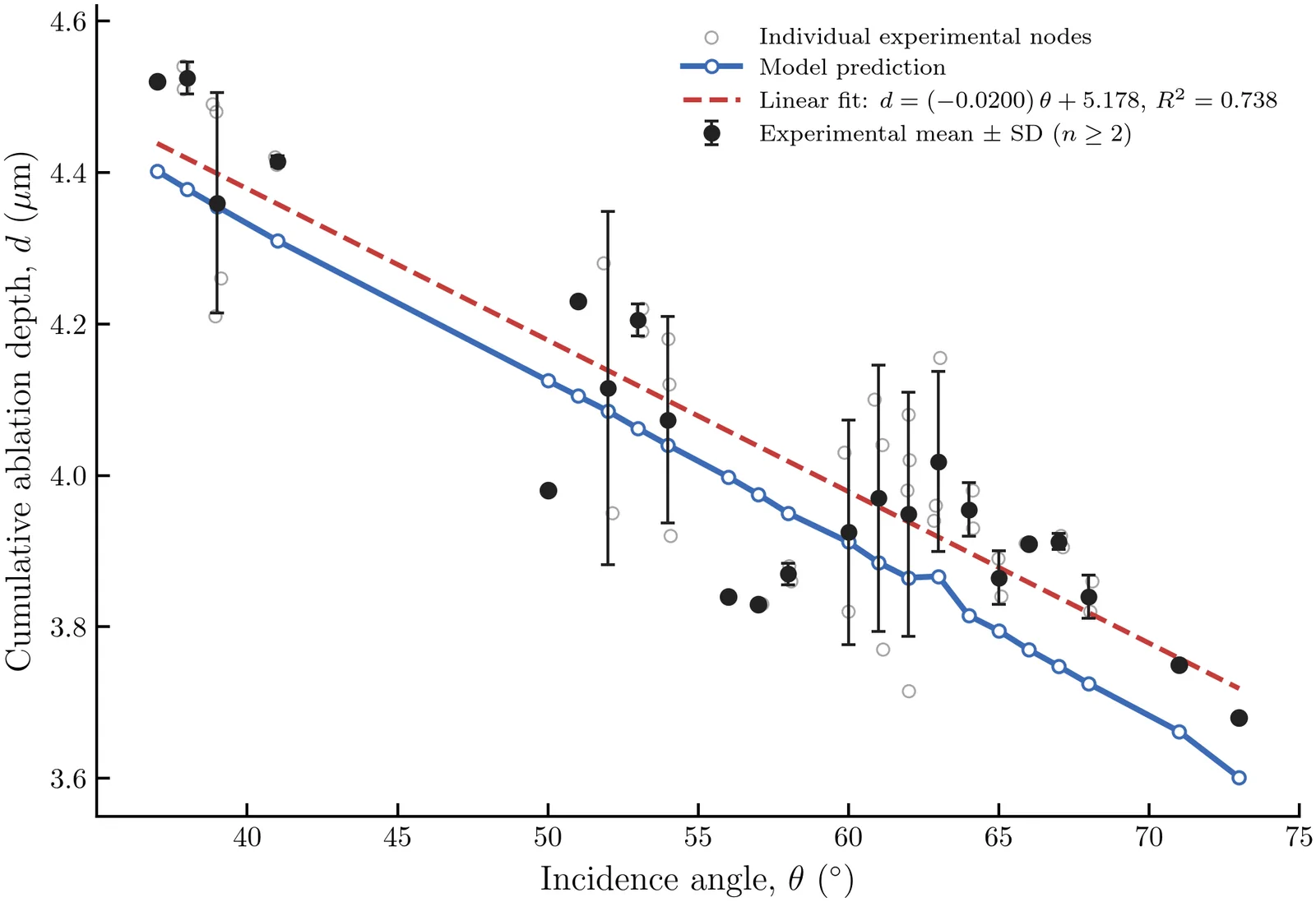
Relationship between cumulative ablation depth and laser incidence angle on the tooth surface. Open gray markers show all 45 experimental nodes; black filled markers and error bars show the mean ± sample standard deviation at incidence angles represented by at least two nodes; the blue curve shows the model prediction; and the dashed red line is the experimental least-squares fit with the explicitly negative slope, d=(−0.0200)θ+5.178 (*R*^2^ = 0.738).

#### 3.3.3 Global tooth surface topography prediction.

Finally, by interpolating the simulated ablation depths of all discrete nodes, a theoretical 3D topography of the entire face gear tooth surface post-ablation was constructed (Fig 13). The material removal rate is inherently lower near the tooth top and the small end (where θ is excessively large), indicating that adaptive parameter compensation is absolutely imperative.

**Fig 13:**
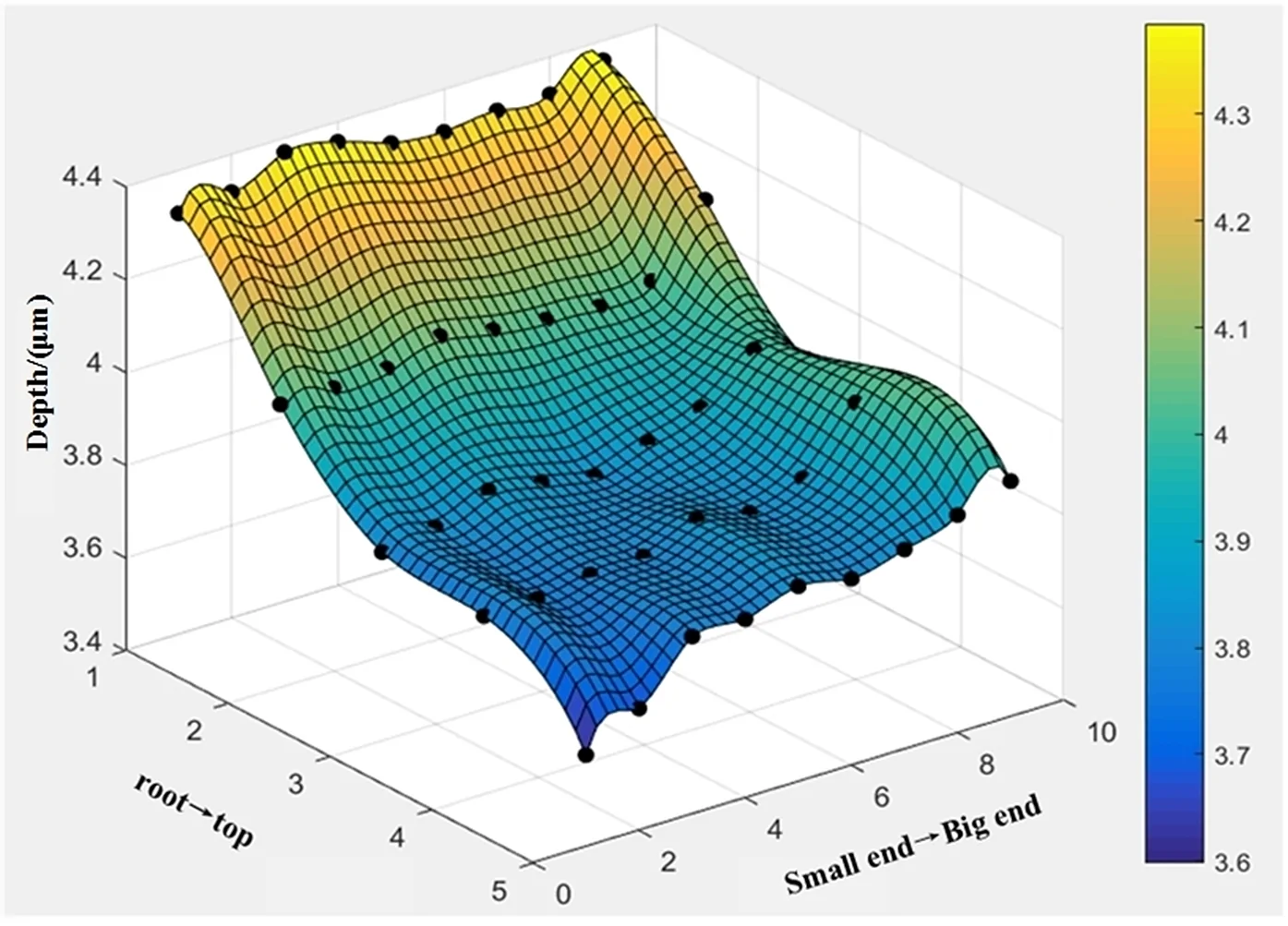
Simulated 3D tooth surface fitting.

## 4 Experimental validation and results discussion

### 4.1 Macroscopic topological validation

After the fs laser precision correction, the global spatial topography of the face gear tooth was measured using a fully automatic Klingelnberg P65 CNC gear testing center. The system was operated under VDI/VDE 2612/2613 Class 1 specifications, and the probe scanned the tooth surface according to the identical 5×9 spatial grid. Cumulative depth was calculated as the local normal separation between registered pre- and post-processing surfaces. Repeatability was evaluated from five repeated registration scans. Including the pre-scan, post-scan, registration, and user contributions, the combined standard uncertainty was


$$\begin{array}{c} u_{c}(d)=\sqrt{u_{pre}^{2}+u_{post}^{2}+u_{reg}^{2}+u_{user}^{2}}. \end{array}$$ (11)

The expanded uncertainty was $U=ku_{c}=0.6\;\mu$m for *k* = 2, corresponding to approximately 95% coverage. This expanded measurement uncertainty is distinct from the error bars in Fig 12, which represent the sample standard deviation among nodes measured at the same incidence angle.

The experimental 3D topography of the ablated tooth surface (Fig 14) exhibits the same overall spatial trend as the theoretical prediction. The measured ablation depth across the entire tooth surface fluctuates within a band of approximately 1 μm.

**Fig 14:**
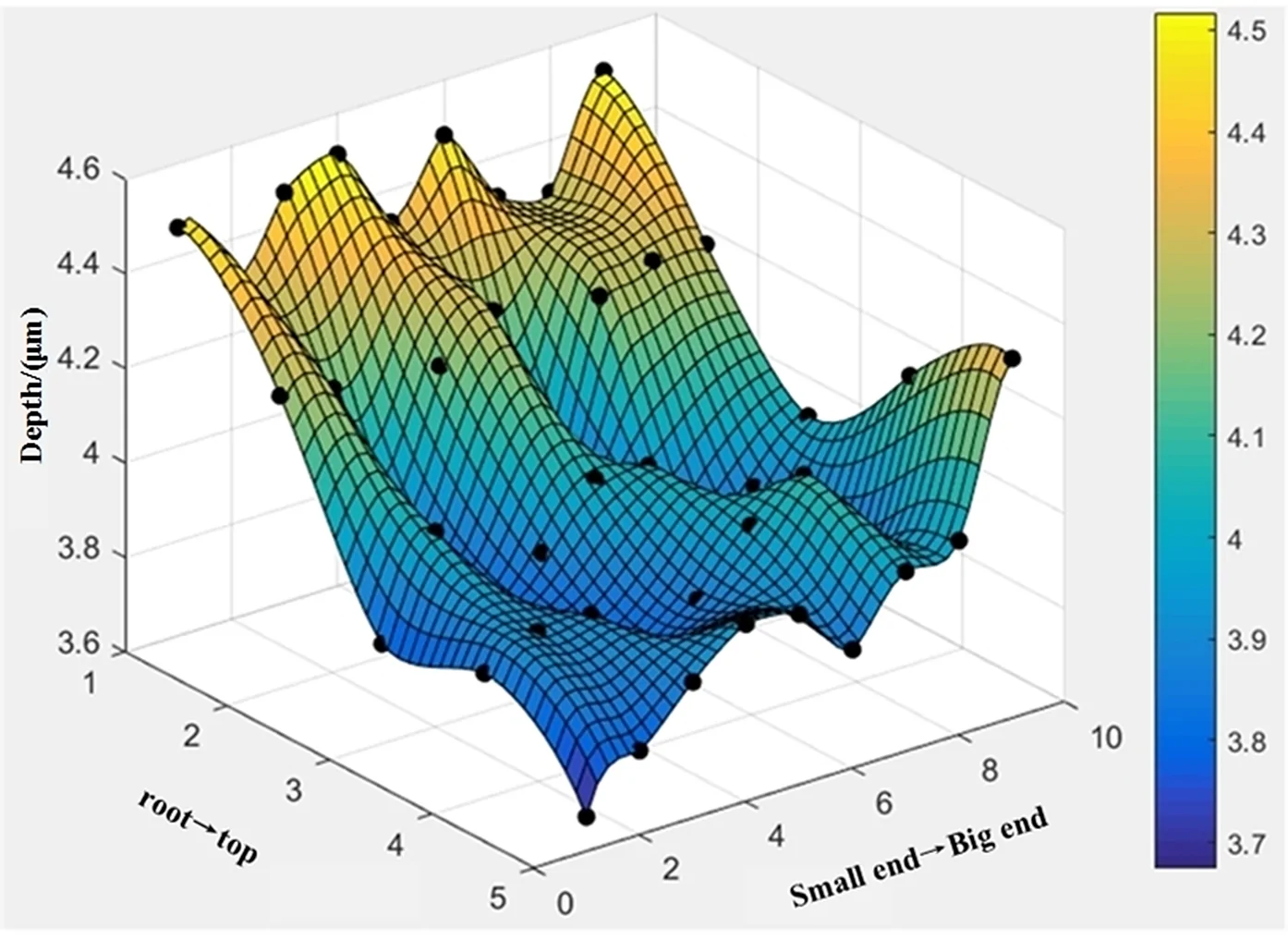
Fitted experimental 3D tooth surfaces.

To quantify the model–measurement differences, an error mapping surface was generated from all 45 grid nodes (Fig 15). Direct recalculation from the node-level data gave a mean absolute error of 0.131μm, a root-mean-square error of 0.137μm, a mean signed error of +0.066μm, and a sample standard deviation of the signed residuals of 0.121μm. The maximum absolute difference was 0.255μm, corresponding to a maximum relative difference of 6.53%. These descriptive differences are smaller than the expanded experimental measurement uncertainty (U=0.6μm); consequently, they should not be interpreted as evidence that sub-*U* discrepancies have been metrologically resolved. A detailed comparison at representative spatial nodes is provided in Table 3; the whole-grid statistics reported here were calculated using all 45 nodes.

**Fig 15:**
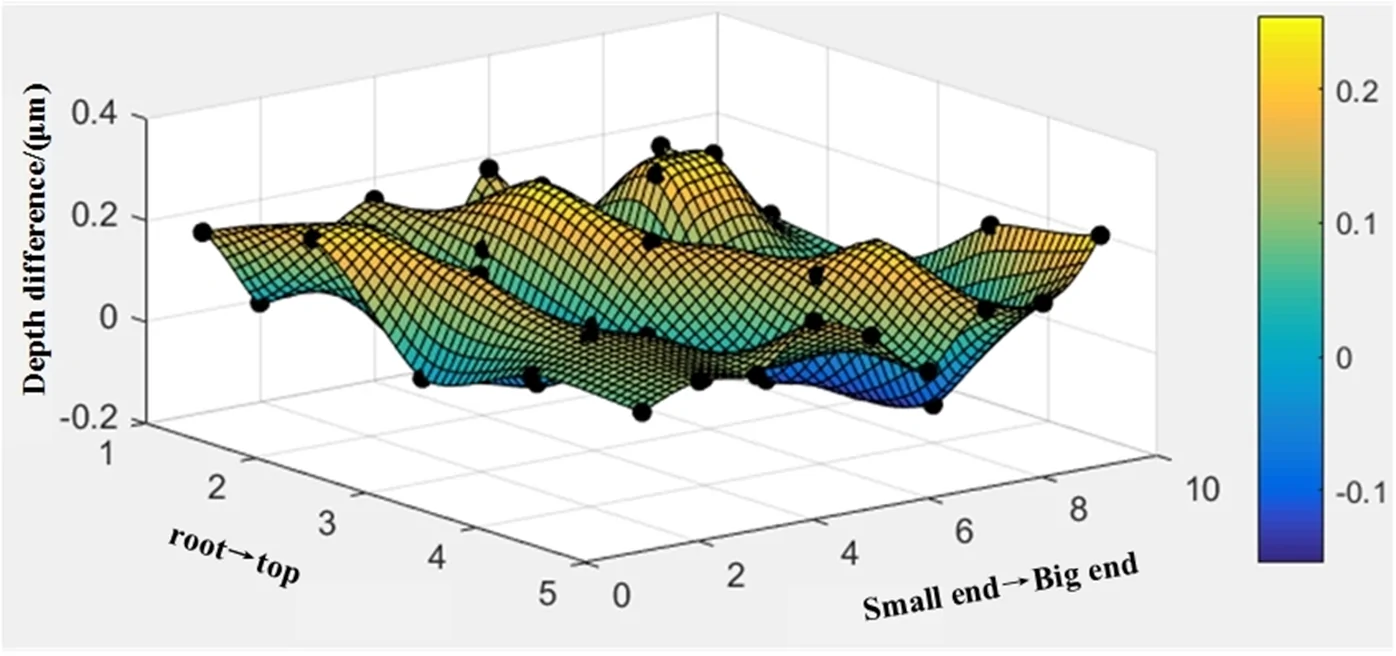
Fitted differential error surface between simulated and experimental ablative pit depths.

**Table 3:** Quantitative comparison between theoretical predictions and experimental measurements of ablation depths at representative spatial nodes.

Representative Node Location	Local Incidence				
Ange (*θ*)	Sim. Depth (μm)	Exp. Depth (μm)	Abs. Error (μm)	Rel. Error (%)	
Top region – Small end	$73^{\circ }$	3.601	3.680	0.079	2.15%
Top region – Big end	$60^{\circ }$	3.912	3.820	0.092	2.41%
**Central concave region**	$63^{\circ }$	**3.900**	**4.155**	**0.255**	**6.53%**
Root region – Small end	$41^{\circ }$	4.310	4.410	0.100	2.27%
Root region – Big end	$37^{\circ }$	4.402	4.520	0.118	2.61%

To isolate the contributions of the two principal geometric-optical effects, nested model variants were evaluated using the same 45 nodes and calibrated parameters (Table 4). The complete model reduced RMSE by 83.8% relative to the baseline model. The progressive error reduction demonstrates that both incidence-angle-dependent Fresnel reflection and dynamic defocusing contribute materially to the prediction, with dynamic defocusing producing the larger incremental improvement under the high-angle conditions.

**Table 4:** Nested-model comparison using the 45 validation nodes.

Model	Dynamic defocusing	Angle-dependent reflection	RMSE (μm)
M0	No	No	0.845
M1	No	Yes	0.620
M2	Yes	No	0.435
M3 (complete)	Yes	Yes	0.137

### 4.2 Mechanism discussion on spatial error distribution

The spatial distribution of the prediction error (Fig 15 and Table 3) exhibits a specific pattern: the discrepancy is slightly more pronounced in the central concave region of the face gear compared with the extremities. Because no time-resolved emission, interferometry, plume imaging, or electron-density measurement was performed, the responsible mechanism cannot be confirmed directly. The observed pattern may be associated with the following coupled effects:

1. **Plasma Plume Confinement and Shielding:** Geometric confinement in the central valley may restrict plume expansion and increase plasma density, which would be consistent with stronger inverse-bremsstrahlung absorption and plasma shielding.
2. **Oxidation in Non-Vacuum Environment:** Residual heat and the interaction of the highly reactive ejected plasma with ambient oxygen can induce mild surface oxidation, altering the actual absorptivity β(θ) dynamically.
3. **Initial Surface Roughness Coupling:** The inherent micro-roughness from prior CNC milling creates micro-scale localized variations in the incidence angle and defocusing state that cannot be fully captured by the macroscopic grid.

Despite these unresolved secondary effects, the dominant nonlinear mechanisms are captured with low whole-grid prediction error, supporting use of the model as a basis for trajectory optimization. Direct plasma diagnostics will be required to test the shielding interpretation.

## 5 Conclusions

The application of femtosecond (fs) lasers for the ultra-precision finishing of aerospace face gears is fundamentally bottlenecked by the nonlinear interactions between the spatially varying light field and the target material. In this study, a comprehensive 3D nonlinear ablation model was established and experimentally validated for 18Cr2Ni4WA alloy steel. The main conclusions are:

1. **Quantification of Spatially Variant Optical Coupling:** The theoretical analysis reveals that the optical reflectivity is highly sensitive to θ, reaching a minimum aggregate reflectivity of 0.567 at an incidence angle of $76^{\circ }$. Concurrently, oblique incidence causes pronounced elliptical beam stretching, geometrically dispersing the local laser fluence.
2. **Mechanism of Dynamic Defocusing on Complex Topologies:** The localized defocusing distance Δf within the ablation crater is directly proportional to tanθ. This effect hyperbolically expands the effective beam radius, causing asymmetrical depth reduction in highly inclined regions.
3. **Macroscopic Topography Prediction:** Comparison with Klingelnberg CNC gear-center measurements showed that the model reproduced the global spatial trend, with an RMSE of 0.137μm and a maximum absolute model–measurement difference of 0.255μm (6.53% relative difference). Because the expanded measurement uncertainty was 0.6μm, these values are interpreted descriptively rather than as metrological proof of submicrometer predictive accuracy.
4. **Insights into Secondary Physical Shielding Effects:** The concentration of residual error in the central concave region is consistent with possible localized plasma-plume confinement and enhanced inverse-bremsstrahlung absorption. Because no direct plasma diagnostics were performed, this interpretation remains a hypothesis for future investigation.

In summary, the established model transitions the fs laser finishing process from empirical trial-and-error to model-driven predictable manufacturing. Future research will focus on integrating this mathematical model into a multi-axis CNC control system to enable real-time, adaptive parameter compensation.
